# Effect of Tff3 Deficiency and ER Stress in the Liver

**DOI:** 10.3390/ijms20184389

**Published:** 2019-09-06

**Authors:** Kate Šešelja, Iva Bazina, Jessica Welss, Martin Schicht, Friedrich Paulsen, Nikola Bijelić, Edi Rođak, Anita Horvatić, Andrea Gelemanović, Martina Mihalj, Mirela Baus Lončar

**Affiliations:** 1 Department of Molecular Medicine, Ruđer Bošković Institute, Bijenička 54, 10 000 Zagreb, Croatia; 2 Institute of Functional and Clinical Anatomy, Faculty of Medicine, Friedrich-Alexander-University Erlangen-Nürnberg, 91051 Erlangen, Germany; 3 Department of Histology and Embryology, Faculty of Medicine, University of Osijek, J. Huttlera 4, HR-31000 Osijek, Croatia; 4 ERA Chaire Team, Proteomics Laboratory, Faculty of Veterinary Medicine, University of Zagreb, Heinzelova 55, 10 000 Zagreb, Croatia; 5 Department of Physiology and Immunology, Faculty of Medicine, University of Osijek, J. Huttlera 4, HR-31000 Osijek, Croatia

**Keywords:** ER stress, trefoil peptide 3, liver, tunicamycin, proinflammatory cytokines

## Abstract

Endoplasmic reticulum (ER) stress, a cellular condition caused by the accumulation of unfolded proteins inside the ER, has been recognized as a major pathological mechanism in a variety of conditions, including cancer, metabolic and neurodegenerative diseases. Trefoil factor family (TFFs) peptides are present in different epithelial organs, blood supply, neural tissues, as well as in the liver, and their deficiency has been linked to the ER function. Complete ablation of *Tff3* expression is observed in steatosis, and as the most prominent change in the early phase of diabetes in multigenic mouse models of diabesity. To elucidate the role of *Tff3* deficiency on different pathologically relevant pathways, we have developed a new congenic mouse model *Tff3^−/−^*/C57BL6/N from a mixed background strain (C57BL6/N /SV129) by using a speed congenics approach. Acute ER stress was evoked by tunicamycin treatment, and mice were sacrificed after 24 h. Afterwards the effect of *Tff3* deficiency was evaluated with regard to the expression of relevant oxidative and ER stress genes, relevant proinflammatory cytokines/chemokines, and the global protein content. The most dramatic change was noticed at the level of inflammation-related genes, while markers for unfolded protein response were not significantly affected. Ultrastructural analysis confirmed that the size of lipid vacuoles was affected as well. Since the liver acts as an important metabolic and immunological organ, the influence of *Tff3* deficiency and physiological function possibly reflects on the whole organism.

## 1. Introduction

Proper protein folding and localization is crucial for protein function. Endoplasmic reticulum (ER) has the main role in the folding and dispatching of secretory and transmembrane proteins to the appropriate destinations. Various conditions, such as increased protein synthesis, decreased ER-associated degradation, disturbed lipid homeostasis, calcium efflux and oxidative stress, can lead to an accumulation of misfolded proteins, and subsequently trigger a condition known as ER stress (ERS) [1]. The adaptive mechanism activated in this situation is known as the unfolded protein response (UPR). The central role of the UPR is to restore protein homeostasis, but in case of a prolonged signal, such as chronic stress and/or severe damage, it can lead to apoptosis. The UPR forms a complex network mediated by three ER membrane-associated proteins, protein kinase R (PKR)-like endoplasmic reticulum kinase (PERK), inositol requiring enzyme 1 alpha (IRE1α) and activating transcription factor 6 alpha (ATF6α). 

These signaling pathways change the expression of numerous genes involved in cytokine production, apoptosis, autophagy, glucose metabolism, folding chaperones and quality control [2]. ERS is recognized as a major pathological mechanism in a variety of conditions, including metabolic and neurodegenerative diseases. Its activation can exacerbate inflammatory and oxidative stress signaling pathways, leading to insulin resistance, fatty liver disease and obesity [3].

Trefoil factory family peptides (TFFs) have been linked to the ER function. *Tff1*-deficient mice have been shown to activate UPR in the stomach tissue [4], whilst *Tff2*-deficient mice exhibit an overexpression of the transporter associated with antigen processing 1 (Tap1), a protein involved in ER-associated degradation as well as the overexpression of chaperone Bcl2 associated athanogene 2 (Bag2) in the pyloric antrum [5]. Screening of the mouse cochlear cDNA library with yeast-two-hybrid assay identified Bcl2-associated athanogene 6 (Bag6/Bat3/Scythe) as a potential Tff3 interactive partner [6]. Bag6/Bat3/Scythe is a protein complex involved in apoptosis and the quality control of membrane proteins [7]. The study done on goblet cells analyzed the impact of lactic acid bacteria on intestinal health, and it demonstrated that tunicamycin (Tm), an inducer of ERS, downregulates the expression of the *Tff3* gene [8]. Furthermore, mice deficient in the protein Oasis, a well-known ERS transducer, exhibit a significantly increased expression of Tff3 in goblet cells [9]. Tff3 (59 amino acid, 7 kDA), predominantly expressed in intestinal goblet cells, has a multifunctional role in protecting mucosa by being involved in cell migration, immune response and apoptosis [10,11]. 

TFFs’ presence in the bloodstream and in various other organs (lung, pancreas, mammary gland, inner ear, lymphoid tissue, and even in the brain) points to their general importance in the organism homeostasis [12]. It has been shown that Tff3 from the liver has neuroprotective and cardioprotective effects [13,14]. Liver Tff3, induced by ischemic brain injury, can pass the blood-brain barrier, and concentrate in an affected region, alleviating the damage. Gene expression of *Tff3* in the liver is significantly downregulated in different mouse models of obesity [15,16], diabesity [17] and liver steatosis [18], conditions that are associated with ER dysfunction. 

Considering its expression in various tissues and its multiple biological roles, including a role in relevant metabolic processes, it is necessary to have a controlled model to elucidate the specific role of Tff3 protein in different parts of organism. Existing *Tff3*-deficient mice [19], generated on a mixed genetic background (C57BL6J/Sv129), have a possible impact of additional mutations and unpredictable genetic combinations on different genetic loci. We have generated a novel congenic strain on a C57BL6/N genetic background to avoid, increased genetic heterogeneity [20], a known problem with heterozygous mixed background strains. Crossing males of the existing mixed background *Tff3^−/−^*/C57BL6/J/SV129 strain [19] with C57BL6/N females led to a novel strain deficient in Tff3 protein, while the rest of the genome is homozygotic, as in the wild type C57BL6/N strain. The C57BL6/J sub-strain has various mutations, including deletion within the nicotinamide nucleotide transhydrogenase (Nnt) gene, which causes an altered metabolic phenotype (https://www.jax.org/strain/000664) [21]. Hence, this C57BL6/N substrain is a more representative model for metabolic studies.

The aim of the present study was to develop a congenic *Tff3*-deficient mouse strain without any additional metabolically-relevant mutations, and to assess the impact of *Tff3* deficiency on disease-related genes (ERS, oxidative stress and inflammation relevant genes) in the liver, as a major metabolic organ. Mice were exposed to acute ERS induced by Tm, a drug that blocks the initial step of *N*-glycosylation, leading to accumulation of misfolded proteins in the ER. Effects of acute ERS were monitored in the liver at the relevant gene/protein expression levels, which were shown to play roles in ERS and oxidative stress signaling, expression of inflammatory genes, and have an impact on the ultrastructural level. 

## 2. Results

### 2.1. Expression of ER Stress Markers

**Figure 1:**
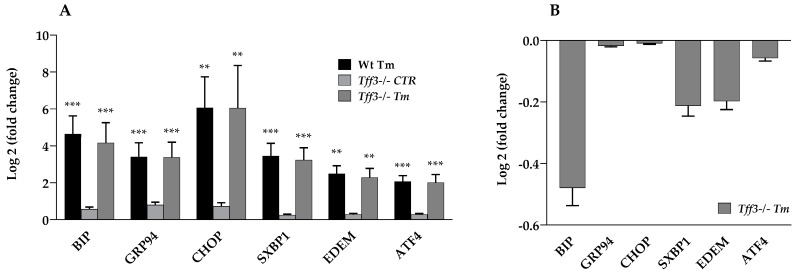
Effect of tunicamycin treatment on the expression of ER stress markers. We performed a quantitative polymerase chain reaction (qPCR) using SYBR green detection chemistry for each group (*n* = 5 animals per group), wherein C_t_ data were analyzed by REST software and presented relative to the Wt control group as log_2_ (fold change) (**A**). Additionally, expression of genes in tunicamycin-treated trefoil factor family 3 (*Tff3*)*^−/−^* mice was expressed relative to Wt tunicamycin-treated mice (**B**). ** *p* ≤ 0.01, *** *p* ≤ 0.001; Wt Tm = wild type tunicamycin-treated mice, *Tff3^−/−^* CTR = *Tff3*-deficient untreated mice, *Tff3^−/−^* Tm = *Tff3^−/−^* tunicamycin-treated mice.

**Figure 2:**
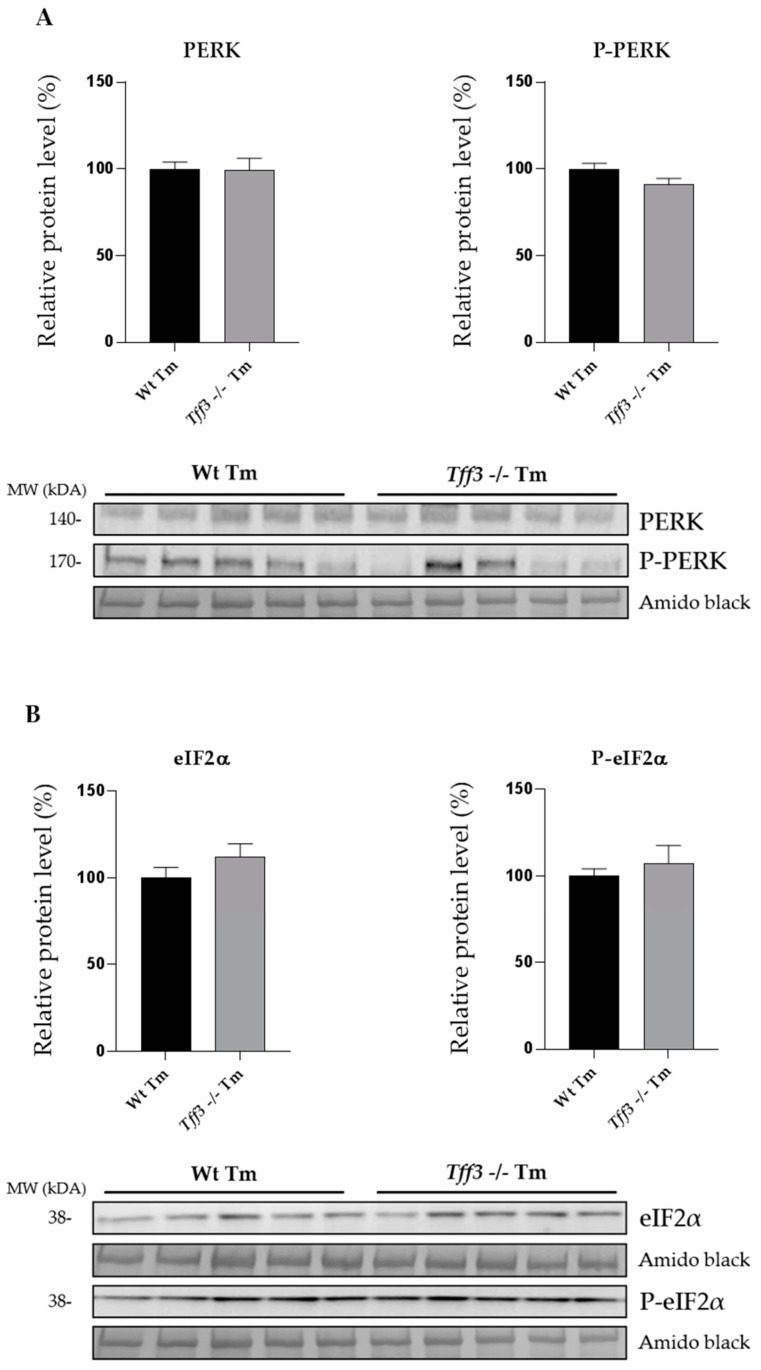
Effect of ER stress on PERK (**A**) and eIF2α (**B**) protein phosphorylation. Relative protein levels of ER stress markers, (PKR)-like endoplasmic reticulum kinase (PERK), p-PERK (**A**) and eIF2α and p-eIF2α (**B**) in liver homogenate of wild type mice and *Tff3^−/−^* mice treated with tunicamycin. Protein level is presented relative to Wt mice as mean ± SEM of specific protein band density normalized with amido black. The difference between the groups was compared by the Student *t*-test. Wt Tm = wild type tunicamycin-treated mice, *Tff3^−/−^* Tm = *Tff3^−/−^* tunicamycin-treated mice.

To analyze the effect of *Tff3* deficiency on ER stress in the liver, we monitored expression levels of the relevant ERS genes (Figure 1) and proteins (Figure 2) in wild type and *Tff3*-deficient, Tm-treated mice (Wt Tm and *Tff3^−/−^* Tm), as well as untreated controls (Wt CTR and *Tff3^−/−^* CTR). Liver tissue was collected 24 h after treatment. Using the SYBR green quantitative polymerase chain reaction (qPCR) method, we compared the levels of several ERS relevant genes: *BIP*, *GRDP94*, *CHOP*, *sXBP1*, *EDEM* and *ATF4*. Comparison of gene expression relative to Wt CTR mice (Figure 1A) showed that Tm treatment activated relevant ERS marker genes, which was statistically relevant and comparable in both mice genotypes. Tm-treated Wt and *Tff3^−/−^* mice showed a similar gene expression level (Figure 1B). *Tff3^−/−^* mice had slightly reduced levels of the ERS marker genes, although this was not statistically significant. Protein levels of PERK and eIF2α and their activated phosphorylated forms were similar in Wt and *Tff3^−/−^* mice (Figure 2). 

### 2.2. Expression of Oxidative Stress Marker Genes 

**Figure 3:**
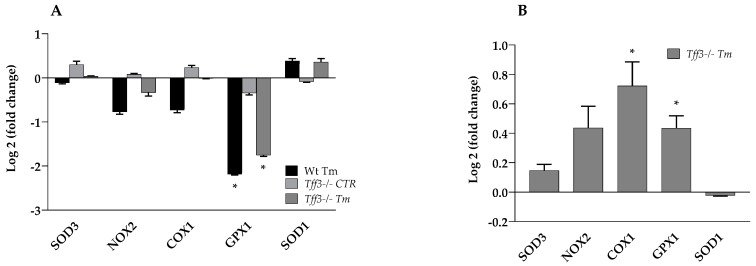
Effect of tunicamycin treatment on the expression of relevant oxidative stress genes in Wt- and *Tff3*-deficient mice. We performed qPCR using SYBR green detection for each group (*n* = 5 animals per group), Ct data were analyzed by the REST program and presented relative to Wt control (**A**). Expression of genes in tunicamycin-treated *Tff3*-deficient mice was expressed relative to Wt tunicamycin-treated mice (**B**). * *p* ≤ 0.05; Wt Tm = wild type tunicamycin-treated mice, *Tff3*^−/−^ CTR = *Tff3* deficient untreated mice, *Tff3^−/−^* Tm = *Tff3^−/−^* tunicamycin-treated mice.

The effect of *Tff3* deficiency on the oxidative stress markers (Figure 3) was monitored in the livers of Wt and *Tff3^−/−^* mice with and without Tm treatment. Gene expression levels relative to untreated Wt mice showed that Tm treatment statistically reduced the expression of *GPX1* in Wt (4.55 fold) and *Tff3^−/−^* (3.5 fold) mice. 

Expression of other marker genes (*SOD3, NOX2, COX1* and *SOD1*) was not affected (Figure 3A). Tm-treated *Tff3^−/−^* mice, compared to Wt Tm-treated mice, revealed a significantly increased expression level of *COX1* (1.65 fold) and *GPX1* (1.35 fold) (Figure 3B). 

### 2.3. Tff3 Deficiency Reduces Cytokine Expression in Acute ERS

**Figure 4:**
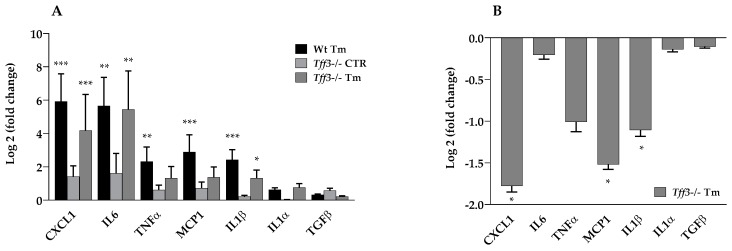
Effect of tunicamycin treatment on the expression of cytokine genes in Wt and *Tff3*-deficient mice. We performed qPCR using SYBR green detection for each group (*n* = 5 animals per group), C_t_ data were analyzed by REST program and presented relative to Wt control as log2 (fold change) (**A**). Expression of genes in tunicamycin-treated *Tff3^−/−^* mice was expressed relative to the Wt tunicamycin-treated mice (**B**). * *p* ≤ 0.05, ** *p* ≤ 0.01, *** *p* ≤ 0.001; Wt Tm = wild type tunicamycin-treated mice, *Tff3*^−/−^ CTR = *Tff3* deficient untreated mice, *Tff3*^−/−^ Tm = *Tff3*^−/−^ tunicamycin treated mice.

**Figure 5:**
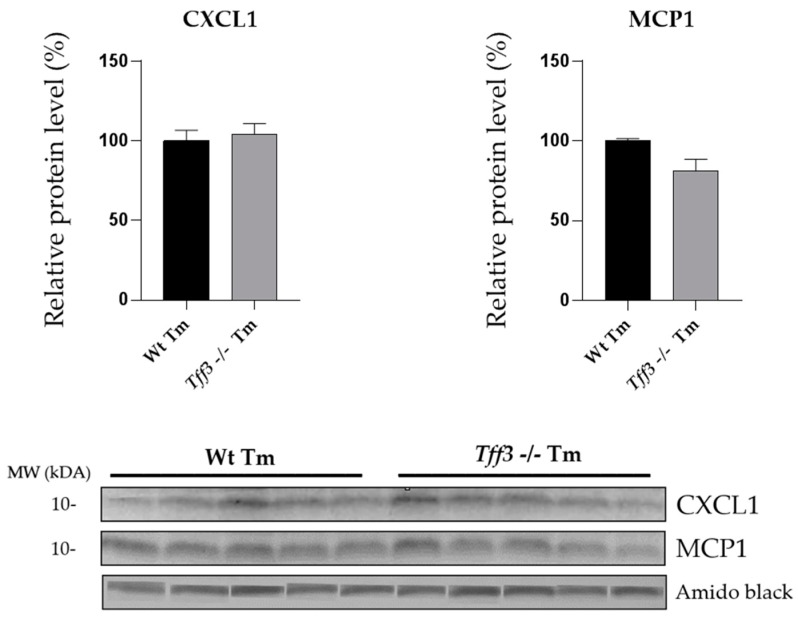
Effect of tunicamycin treatment on the expression of CXCL1 and MCP1 protein in Wt and *Tff3*-deficient mice. Protein level of CXCL1 and MCP1 was determined by Western blot in liver homogenate of WT (C57BL6/N) and *Tff3^−/−^* mice treated with tunicamycin. Protein level is presented relative to Wt mice as mean ± SEM of specific protein band density normalized with amido black. The differences between groups were compared by the Student *t*-test. Wt Tm = wild type tunicamycin-treated mice, *Tff3^−/−^* Tm = *Tff3^−/−^* tunicamycin-treated mice.

Both Wt and *Tff3^−/−^* mice showed an upregulation of liver cytokine gene expression upon ERS induction: *CXCL1* (60.67 fold), *IL6* (50.25 fold), *MCP1* (7.40 fold), *IL1β* and *TNFα* (5 fold). *IL1α* and *TGFβ* were not affected (Figure 4A). *Tff3^−/−^* Tm mice had a statistically relevant reduced expression of *CXCL1* (3.43 fold), *MCP1* (2.87 fold) and *IL1β* (2.15 fold) compared to Wt Tm mice (Figure 4B). MCP1 and CXCL1 protein levels were monitored by Western blot analysis (Figure 5), but protein levels did not reveal the difference observed at the mRNA level (Figure 4B).

### 2.4. Shotgun Proteomic Analysis of Liver Proteome ERS Provoked WT and Tff3 Deficient Mice

**Figure 6:**
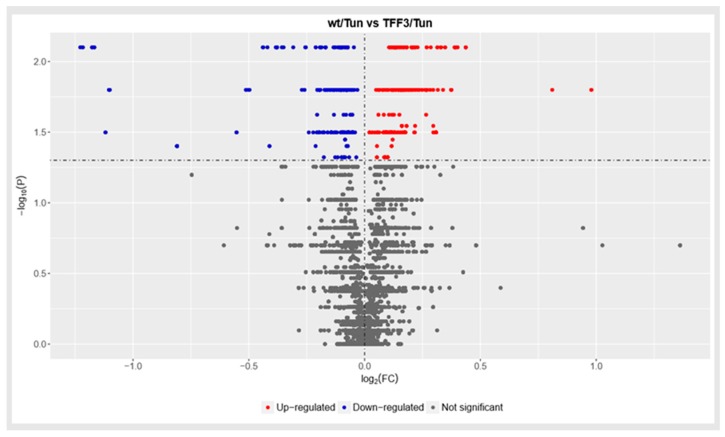
Volcano plot, plotting significance (−log(P)) on the y-axis, versus fold change (log2FC) on the *x*-axis.

Comparing the global protein expression levels of Tm-treated Wt and *Tff3*-deficient mice, a difference between the groups is shown (Figure 6). *Tff3^−/−^* mice livers had changed the expression of antioxidative proteins involved in the resolution of Tm-induced oxidative stress. Metallothionein-1 (MT1) and metallothionein-2 (MT2) had a 2-fold increase in level, while glutathione S-transferase pi 1 (GSTP1) and glutathione S-transferase I pi 2 (GSTP2) had a 3-fold level reduction. Applying an additional statistical approach using false discovery rates (FDR) that compensated for large data sets, these changes did not reveal any relevant statistical reliability (Data in Supplement: Table S1).

### 2.5. Histological and Ultrastructural Changes

**Figure 7:**
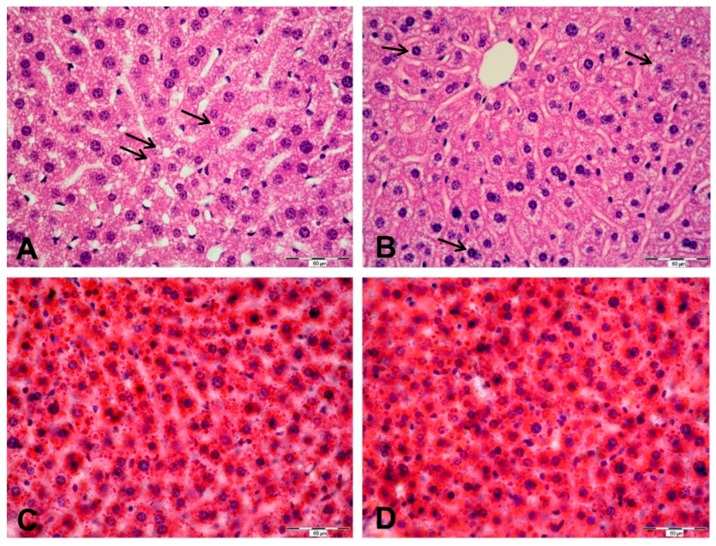
Liver tissue of wild type (**A**,**C**) and *Tff3* knock-out (**B**,**D**) tunicamycin-treated mice stained using hematoxylin-eosin (**A**,**B**) and oil red O (**C**,**D**). Numerous small lipid droplets (marked with black →) are visible in the cytoplasm of hepatocytes stained using hematoxylin-eosin. There is no apparent morphological difference in fatty changes between the two groups. On oil red O-stained slides, lipid droplets stained intensively red are abundant in hepatocyte cytoplasm across the slide. There is no apparent morphological difference in fatty changes between the two groups. Scale bar: 60 µm.

Hematoxylin-eosin stain revealed an extensive fat accumulation in the form of numerous small lipid droplets in hepatocytes, both in Wt- and *Tff3^−/−^* Tm-treated mice (Figure 7). Almost all of the examined hepatocytes were affected. No histomorphological differences were observed between the two groups. No fibrosis or signs of inflammation were detectable. Cryosections of the same livers stained with Oil Red O supported this result, revealing numerous lipid droplets in hepatocytes stained intensively red. Semi-quantitative analysis of the share of lipid droplet area showed no statistically significant difference between the groups; median share of lipid droplets for the Wt and *Tff3^−/−^* groups was 56.42 and 59.63%, respectively (*p* = 0.75, Mann-Whitney U test). 

**Figure 8:**
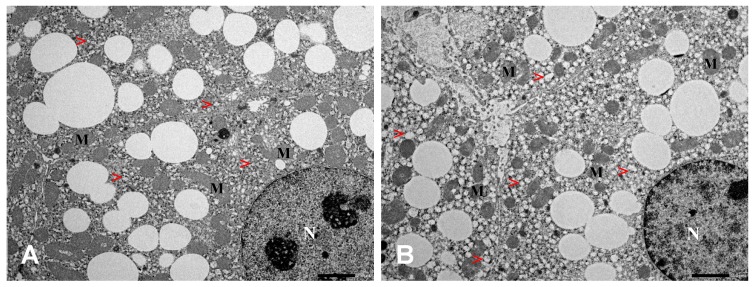
Liver tissue of wild type (**A**) and Tff3 knock-out (**B**) tunicamycin-treated mice analyzed by transmission electron microscopy (TEM); (M) mitochondria, (N) nucleus, (>) lipid droplets. The size of lipid droplets in hepatocytes appeared to be increased in *Tff3^−/−^* mice compared to wild type mice. There was no difference in the ultrastructure of hepatocytes including cell bodies and cell organelles. Scale bar: 2 µm.

Analysis of ultrastructure (Figure 8) showed no changes in the morphology of the hepatocytes or capillaries. Liver cells demonstrated normal cell bodies with dense and round nuclei and moderate amounts of cytoplasm. No accumulation of autophagosomes, lysosomes or peroxisomes was detected. Kupffer cells were counted, and a comparable amount of liver macrophages were found in control and *Tff3^−/−^* mice. General morphology of mitochondria appeared to be normal, including inner and outer mitochondrial membranes. Mitochondria were often surrounded by rough endoplasmic reticulum (RER) in both *Tff3^−/−^* and Wt animals. Fat vacuoles in the cytoplasm of the hepatocytes were found to be visibly enlarged in *Tff3*^−/−^ mice (Figure 8). 

## 3. Discussion

Unresolved ERS underlies various pathological changes in different organs, and its activation is associated with metabolic diseases like obesity, type 2 diabetes (T2D) and nonalcoholic fatty liver disease (NAFLD) [22,23,24]. Exact mechanisms of interaction between ERS and metabolic disruption are not yet fully understood. Liver, as an important metabolic organ, regulates blood glucose and synthesizes fatty acids from glycolytic products in the process of *de novo* lipogenesis [25]. Complete loss of *Tff3* expression was the most prominent expressional change during the early stages of diabetes in the liver of the polygenic mice model of diabesity [17], and since then its role in relevant metabolic processes has emerged [15,16]. Hence, our goal was to investigate the impact of *Tff3* deficiency on relevant ER, oxidative stress and inflammation pathways, which are the key mechanisms in the development of pathological changes in the liver. Since Tffs-deficient mouse models show evidence of disturbed protein processing and possible interaction with chaperones [4,5,6,7,8,9], we have compared the effect of acute ERS in the liver of novel, congenic, *Tff3*-deficient mice and relevant wild type control. ER stress was induced by Tm, a drug shown to successfully mimic hepatic steatosis [26]. Activation of the UPR was monitored by qPCR analysis of downstream targets of UPR. Analyzed genes are common genes used to examine UPR activation: endoplasmic reticulum chaperone BIP (*BIP*), activating transcription factor 4 (*ATF4*), DNA damage-inducible transcript 3 (*CHOP*), ER degradation enhancing alpha-mannosidase like protein 1 (*EDEM1*), heat shock protein 90 (*GRP94*) and spliced X-box binding protein 1 (*sXBP1*) [27]. Upon perturbance in protein folding, BIP dissociates from the three key master regulators of UPR, i.e., IRE1, PERK and ATF6, and consequently activates them. ATF4 and CHOP are transcription factors activated in the PERK signaling pathway. ATF4, amongst other functions, plays a distinct role in autophagy resulting from Tm-induced ERS [28], while CHOP has a complex proapoptotic role [24]. In response to ERS, IRE1 cleaves XBP1, resulting in sXBP-1, an active UPR transcription factor which exerts strong pro-survival effects under various conditions [29]. EDEM1 is an ER-associated protein degradation (ERAD) chaperone [30], and GRP94 performs unique chaperone functions in the ER [31]. Additionally, we analyzed UPR activation on the protein level by measuring PERK activation with a phospho-specific PERK antibody, as well as the phosphorylation of its downstream target eukaryotic translation initiation factor 2 alpha (eIF2α). 

Results showed that an activation of analyzed UPR markers does not statistically differ in 7-week-old male *Tff3^−/−^* mice, compared to the relevant wild type control on transcriptional and protein levels (Figure 1). It seems that *Tff3* deficiency does not have a significant effect on the expression of the key components of the UPR pathway in Tm-induced acute ERS.

Oxidative protein folding in endoplasmic reticulum is an important resource of reactive oxygen species (ROS) production. Some evidence suggests that endoplasmic reticulum has antioxidant protection that may not be sufficient to relieve the oxidative stress caused by increased protein folding [32]. Tff3 was linked to oxidative stress in human tumor cells, where it was upregulated in the late phase of response to oxidative stress caused by X-rays [33]. Glutathione peroxidase 1 (GPX1) is an antioxidant enzyme [34] expressed ubiquitously in the mitochondria and cytoplasm of many tissues. Wild type and *Tff3^−/−^* Tm-treated mice show a statistically significant reduction in the expression of *GPX1* (4.55 and 3.5 fold, respectively) compared to Wt untreated mice (Figure 3A). *Tff3^−/−^* Tm-treated mice have a slightly but significantly higher expression level of *GPX1* (1.35 fold) relative to Wt Tm-treated mice (Figure 3B). Previous reports showed no significant difference in liver *GPX1* expression between WT and *Tff3^−/−^* mice of mixed background in control condition similar as in this new strain background [35]. GPX activity in NAFLD livers compared to control is significantly higher [36] and the severity of disease is correlated with the decreased activity of GPX [37].

Cyclooxygenase 1 (COX1) is an enzyme located in the endoplasmic reticulum and nuclear envelope of a wide variety of cells and tissues. It forms prostaglandins via arachidonic acid oxygenation by a free radical mechanism [38], hence, it may produce toxic oxygen species [39]. Expression of COX1 is essential for hepatic homeostatic maintenance [40]. Both Tff3 and COX-1 take part in the protection of intestinal mucosa and promotion of cell migration [41,42]. 

While Tff3 regulates gastric epithelial restitution via a COX1 independent pathway [43], the activation of cellular invasion by Tff3 is mediated by a COX1 dependent signaling pathway [42]. *Tff3^−/−^* untreated animals show a slightly higher expression of *COX1* than untreated wild type animals (Figure 3A), and Tm-treated animals reveal a significant difference (Figure 3B), indicating the impact of *Tff3* deficiency on *COX1* expression in acute ERS. Xiao et al. found that deficiency or inhibition of COX-1 during acute liver injury, increased hepatic oxidative stress, inflammation and apoptosis in mice. Specifically, they showed a higher expression of MCP-1 and IL-1β [40]. The similar situation regarding the expression of previously mentioned genes is in accordance with our results (Figure 3A,B and Figure 4A,B). Tff3 protein has been found to be upregulated in forms of intestinal inflammation, which is the site of its predominant expression. It is also synthesized in lymphoid tissues (spleen, thymus, lymph nodes, bone marrow), and it stimulates migration of monocytes, which suggests a potential role in the immunological response to tissue injury [44]. Furthermore, it is demonstrated that *Toxoplasma gondii*-infected, *Tff3*-deficient mice, show a significantly down-regulated expression of *IFN γ, IL-12, IL-1β* and *TNFα*, involved in the inflammatory response [45]. 

ERS leads to the production of various pro-inflammatory molecules [46] and Tm treatment causes a strong increase in the expression of *CXCL1* (60.67 fold), *IL6* (50.25 fold), *MCP1* (7.40 fold) in the liver of both Wt and *Tff3*^−/−^ mice. *Tff3*^−/−^ mice have significantly reduced levels of several proinflammatory cytokines (downregulated *CXCL1*: 3.43 fold; *MCP1*: 2.87 fold and *IL-1β*: 2,15 fold) compared to the wild type (Figure 4B), indicating a diminished inflammatory response in the case of *Tff3* deficiency. As mentioned before, *IL-1β* was also found to be downregulated in the ileum of *Tff3*^−/−^ mice infected by *Toxoplasma gondii* [45]. 

Immune cells and hepatic inflammation have a central role in the pathogenesis of metabolic disease, and chemokines are crucial immune cells in the regulation and activation of hepatocytes and circulating immune cells [47]. MCP1 is an important CC-chemokine, involved in the recruiting and activation of immune cells to the site of tissue injury [48]. Transgenic mice overexpressing MCP1 demonstrate insulin resistance and increased hepatic triglyceride content in adipose tissue, suggesting a significant role of MCP1 in the hepatic steatosis of early liver injury [49]. Mandrekar et al. show that *MCP1^−/−^* mice have reduced steatosis in alcoholic liver injury, and they also demonstrate that MCP1 in the liver regulates macrophage activation and proinflammatory cytokines [50]. 

CXCL1 is a chemokine expressed in macrophages, epithelial cells and neutrophils with a key role in neutrophil recruitment and activation [51]. It is known that hepatic neutrophil infiltration, a symptom of steatosis, is linked with various liver pathological conditions via mechanisms of generating reactive oxygen species (ROS) and the production of pro-inflammatory mediators [52]. Wang et al. show that inhibition of CXCL1 did reduce hepatic neutrophil infiltration, and consequently ameliorated steatohepatitis in a mice model of disease. IL-1β signaling can exacerbate an accumulation of cholesterol [53] and triglyceride [54] in the liver. Moreover, *IL-1β*-deficient mice during NAFLD had reduced inflammation and steatosis in the liver [55]. 

The changes noticed at the level of gene expression were not followed at the protein level. Western blot showed unchanged levels of MCP1 and CXCL1 protein. This could be explained by the differential dynamics of the mRNA and the complex relationship in concentrations of mRNAs and proteins during misfolding stress, having a peak between 2–8 h upon ERS induction [56], and we have monitored the effects 24 h upon treatment. Thus far the role of Tff3 protein in immune processes is not thoroughly known. Our data suggest that *Tff3*-deficient mice have less ability to start a pro-inflammatory cascade in acute ERS. 

Total proteome analysis distinguishes two different sample types. Changes were found at the level of antioxidative proteins that are involved in the resolution of Tm-induced oxidative stress in the liver of *Tff3*-deficient mice. MT1 and MT2 have a 2-fold increase in levels, while GSTP1 and GSTP2 have a 3-fold level reduction. Metallothioneins are cysteine-rich, heavy metal-binding proteins with antioxidant properties [57]. They are involved in DNA protection, oxidative stress, angiogenesis and apoptosis [58], and hepatic metallothionein expression seems to have a protective role in liver pathology [59]. Decreased expression of MT1 and MT2 in the liver is associated with liver steatosis in high fat diet-induced, obese mice [60]. In line with our findings, Kondoh et al. found that the Tm treatment enhanced the hepatic MT levels in C57BL/6J mice [61]. 

Metallothionein-1G overexpression in human colorectal cancer cells HT-29 considerably enhances the induction of Tff3 [62]. Gluthatione *S*-transferases play a role in cell defense against oxidative stress and are involved in liver pathologies [63]. GSTP was identified as an ER-resident protein, where it demonstrates both chaperone and catalytic functions. Glutathionylation of multiple ER-resident proteins is vital for ER homeostasis and UPR, and it impacts cell sensitivity to ERS-inducing drugs, like Tm [64]. It can also interfere with ERS-induced apoptosis.

Applying a specific large database statistical approach called false discovery rate (FDR) analysis that normalizes large data sets, we observed no statistically significant changes between Tm-treated Wt and *Tff3*-deficient mice 24 h upon treatment. 

Tm-induced ERS causes hepatic steatosis via the upregulation of a very low-density lipoprotein receptor (VLDLR) and consequently increases lipoprotein delivery to the liver [65]. It also disturbs lipid metabolism and the accumulation of fat in a form of numerous small lipid droplets in hepatocytes that is evident in Wt and *Tff3*-deficient mice liver (Figure 7). Our histological approach applying hematoxylin-eosin and oil red O staining did not reveal obvious differences between the groups. However, ultrastructural analysis revealed that ERS provoked *Tff3*-deficient mice have slightly enlarged fat vacuoles compared to wild type mice (Figure 8). This finding is consistent with results from a former study where Guillén et al. showed a decreased *Tff3* gene expression in mice with a stronger extent of liver steatosis [18]. Our findings in the liver of mixed background *Tff3^−/−^* mice on a normal diet show that fat vacuole formation was also affected [35]. Ultrastructural analysis of the liver tissues show no difference in a number of immunologically relevant cells, and no difference in the general morphology of mitochondria, hepatocyte cell bodies, cytoplasm or capillaries between Tm-treated *Tff3*^−/−^ mice and Wt control mice.

## 4. Materials and Methods 

### 4.1. Experimental Animals 

A novel congenic Trefoil factor family 3 (*Tff3*)-deficient mouse strain on C57BL6/NCrl (Charles River) background was developed from an existing *Tff3*-deficient mixed background strain (C57BL6/J/SV129) [19] using a ‘speed congenics’ approach. Mixed background *Tff3^−/−^* males were crossed with C57BL6/NCrl females, and heterozygote *Tff3^−/−^* males were detected and analyzed for 500 SNP polymorphisms (ENVIGO) to identify the *Tff3-/+* male closely resembled to the C57BL6/NCrl strain. This approach was used for five consecutive back crossings to identify the male carrier with 100% similarity to the C57BL6/NCrl strain regarding relevant SNP loci. Resulting offspring were set up to mate according to a brother x sister scheme, homozygous *Tff3^−/−^* male and female mice (F0 generation) were identified and used to start breeding a new *Tff3^−/−^*/C57BL6/NCrl strain. This new congenic T*ff3^−/−^* mice and wild type mice (C57BL6/NCrl) differ only in the Tff3 region and a small surrounding fragment inherited from the embryonic cell of SV129 strain, while the rest of the genetic loci are homozygous. Thus, we can be more confident that the observed phenotype is a consequence of inactivated Tff3 protein. Male mice of *Tff3^−/−^*/C57BL6/NCrl and the related C57BL6/NCrl wild type strain were used for all further experiments. Animals were kept under standard care conditions. Experimental animal manipulations and procedures performed in course of the Croatian Science Foundation grant IP-06-2016-2717 were approved by the local ethical committee. 

### 4.2. Inducing ER Stress

Acute ERS was induced by a single injection Tm treatment. Stock Tm solution was prepared in sterile dimethyl sulfoxide (DMSO) (10 µg/mL) and diluted with 150 mM dextrose. Tm (3 µg of Tm/g of mouse body weight in a 300 µL final volume) was administered intraperitoneally to *Tff3*-deficient and wild type mice that were seven weeks old. Control animals received 300 µL of 150 mM dextrose. Mice were sacrificed and their tissue was harvested for further analysis after 24 h. During that time food and water were available *ad libitum*.

### 4.3. Quantitative PCR

Total RNA was isolated from the livers of *Tff3^−/−^* mice and Wt controls using an RNeasy Mini Kit (Qiagen, Hilden, Germany), according to the manufacturer’s instructions. 1.5 µg of RNA was transcribed into cDNA with a High-Capacity cDNA Reverse Transcription Kit (Applied Biosystems, Dreieich, Germany). We performed a quantitative polymerase chain reaction (qPCR) using SYBR Green I (Invitrogen, Waltham, MA, USA) detection chemistry and specific primers (Table 1) on the StepOnePlus™ qPCR System (Applied Biosystems). The cycling conditions comprised three min polymerase activation at 95 °C and 40 cycles, including 95 °C for 1min, annealing temperature specific for each primer pair (Table 1) for 30 s, and elongation at 72 °C for 30 s. A single product amplification was confirmed by melting curve analysis. Gene expression was analyzed by REST © software (ΔΔCt method) and normalized to stable housekeeping genes, β-actin (ACTβ) and β2-microglobulin (B2M). Changes were represented as log_2_ (fold change).

### 4.4. Western Blot

Liver proteins were isolated from Tm-treated *Tff3^−/−^* (*n* = 5) and Wt mice (*n* = 5) using RIPA buffer (50 mM TRIS HCL, pH8, 150 mM NaCl, 1 mM EDTA, 1% NP40, 1% sodium deoxycholate, 0.1% SDS) supplemented with phosphatase and protease inhibitors (Roche, Basel, Switzerland). Total protein concentration was determined by a BCA protein assay kit (Pierce, ThermoFischer, Waltham, MA, USA) and 25 µg of proteins per lane was separated by Sodium dodecyl sulfate polyacrylamide gel electrophoresis (SDS-PAGE). Proteins were transferred to a nitrocellulose (ERS markers) or PVDF membrane (cytokines), blocked with 5% BSA in TBS-T and incubated overnight at 4 °C with primary antibodies. The following monoclonal antibodies were used: Anti-(PKR)-like endoplasmic reticulum kinase (PERK) (#3912), anti-phospho-PERK (#3179), polyclonal anti-eIF2α (#9722) and anti-phospho eIF2α (#9721) antibodies from Cell Signaling Technology at dilution 1:1000 in 5% BSA/TBST. Cytokines were detected using rabbit anti-MCP-1 at 1:500 dilution (A00056-4) and anti-CXCL1 at 1:8.000 dilution (A00533) (Boster Biological Technology, Pleasanton, CA, USA) by incubation overnight at 4 °C. The following secondary antibodies were used for detection, that is, goat anti-rabbit IgG-HRP antibody (#170-6515; Bio-Rad, Hercules, CA, USA) or goat anti-rabbit IgG -HRP (#170-6515; Bio-rad). The chemo luminescence signals were detected (Alliance 4.7 Imaging System UVITEC, Cambridge) and analyzed with Image J. Amido Black staining was used as loading control and for the normalization of the bands. 

### 4.5. Proteomic Analysis

#### 4.5.1. Protein Extraction and Sample Preparation

Proteins from the tissue were extracted using a Minute Kit according to manufacturer’s instructions. The total protein concentration in lysates was determined using a BCA protein Assay (Thermo Scientific, Rockford, Waltham, MA, USA). The internal standard was prepared by pooling all samples used in the study (equal protein amounts). TMT labeling was performed as described [66]. Shortly, 30 µg of proteins was diluted with 0.1 M triethyl ammonium bicarbonate (TEAB, pH 7.8 (slightly alkaline) up to a final volume of 50 µl. Samples were reduced (2.5 µL of 200 mM DTT, 1 h at 55 °C, alkylated (2.5 µL of 375 mM IAA, 30 min at room temperature in the dark) and acetone-precipitated (six volumes of ice-cold acetone, −20 °C overnight). After centrifugation (8000× *g*, 10 min at 4 °C), 50 µg of pellets were dissolved in 50 µL of 0.1 M TEAB and trypsin digested (1:40, *w*/*w*) at 37 °C overnight. Tryptic peptides were labeled using TMT tenplex reagents (Thermo Scientific, Rockford, Waltham, MA, USA) which were prepared according manufacturer’s instructions. An amount of 19 µL was added to each sample for the labeling reaction (60 min, RT), which was quenched using 5% hydroxylamine (15 min, RT). Four TMT-labeled peptide samples were combined with the internal standard into the new tube, aliquoted, dried and stored at −80 °C for further analysis. 

#### 4.5.2. LC-MS/MS Analysis

High-resolution LC-MS/MS analysis was performed for protein identification and relative quantification as previously reported [66]. In short, prior to analysis, dried TMT-labeled peptides were dissolved in loading buffer (2% ACN in 0.1% FA) and an amount of 1 μg was desalted on the trapping column by employing the Ultimate 3000 RSLCnano system (Dionex, Germering, Germany). Peptides were then separated using PepMap™ RSLC C18 (50 cm × 75 μm ID) column during 2 h linear gradient of 5–35% buffer B (0.1% FA in 80% ACN) at a flow rate of 300 nL/min. Nanospray Flex ion source and stainless steel emitter (New Objective, Woburn, MA, USA) was used. The ionization voltage was set to 2.1 kV, and the ion transfer tube temperature at 250 °C. DDA was performed in a positive ion mode using the Top 8 method, using Q Exactive Plus (Thermo Scientific, Bremen, Germany). Full scan FTMS spectra were acquired in a mass range *m/z* 350.0 to *m/z* 1900.0 with a resolution of 70,000, AGC target 1 × 10^6^, and the maximum injection time 110 ms. For MS/MS scan, step collision energy was set to 25, 35 and 40% NCE with resolution 17,500 and AGC target 2 × 10^5^. An isolation window of ± 2.0 Da was applied to isolate precursor ions with dynamic exclusion of 30 s. 

#### 4.5.3. Data Analysis 

A database search was performed using the SEQUEST algorithm implemented into Proteome Discoverer (version 2.3., Thermo Fisher Scientific) against *Mus musculus* FASTA files (NCBI database, downloaded 7/12/2017, 46105 entries) according the following parameters: two trypsin missed cleavage sites, precursor and fragment mass tolerances of 10 ppm and 0.05 Da, respectively; carbamidomethyl (C) as a fixed peptide modification, oxidation (M) and TMT sixplex (K, peptide N-terminus) as dynamic modifications. The FDR for peptide identification was calculated using the Percolator algorithm within Proteome Discoverer workflow, based on the search results against a decoy database. At least two unique peptides and 5% FDR were set to obtain reliable protein identification. For the reporter-based relative quantification, an internal standard was used to compare the data between the experiments. 

All statistics was performed using R v3.2.2 [67]. Proteins with 100% missing data were removed from the analysis. Sample outliers were detected per each group for each of the proteins using the Dixon’s test from R package outliers v0.14 [68]. If any sample outlier was significant (p > 0.05) it was removed from further analysis. To test the difference in protein abundance between groups, the Wilcoxon-Mann-Whitney test was performed. Fold change between two groups was calculated as mean (Group2)/mean (Group1) and expressed on log2 scale. PCA and volcano plots were designed using R package ggplot2 v3.1.1 [69]

### 4.6. Histology

Liver tissue was fixed in 4% paraformaldehyde; half of it was paraffin-embedded, and the other part cryo-protected in sucrose, frozen in liquid nitrogen and stored at −80 °C. Paraffin-embedded tissues were cut into 5 µm sections using a rotary microtome (Slee CUT 4060, Slee, Mainz, Germany), deparaffinized and used for hematoxylin-eosin staining. Frozen tissues were cut on a cryostat (Leica CM 3050 S, Leica, Wetzlar, Germany) into 20 µm-sections and stained with oil red O stain. The Olympus^®^ BX-50 light microscope (Olympus, Tokyo, Japan), Olympus^®^ C-5050 digital camera and QuickPHOTO Pro software (Promicra s.r.o, Prague, Czech Republic) were used to obtain digital photographs. Images of oil red O-stained tissue were processed and analyzed in FIJI software (FIJI is Just ImageJ), a distribution of ImageJ2 open-source image processing software (Schindelin et al., 2012, 2015). Images were transformed into black and white masks by adjusting the color balance, using Color transformer 2 plugin and image thresholding on the channel containing a signal from red-stained lipid droplets. Lipid droplet area and total tissue area were measured in FIJI, and share of lipid droplet area was calculated [70,71]. Blood vessels larger than liver sinusoids and artifacts were subtracted from the total tissue area, if present on digital photographs. Three representative photographs were taken for each animal under 40× objective, and after measurements, average values were calculated for each animal and used for statistical analysis.

### 4.7. Ultrastructure 

*Tff3*-deficient mouse strains on C57Bl6/NCrl were perfused with 4% PFA, and tissues were immersion fixed immediately after removing in Ito’s fixative. Liver was post-fixed in OsO4, dehydrated in a graded ethanol series, and whole tissues were embedded in Epon resin. Tissue sections (1 µm thick) were cut with an ultramicrotome (Ultracut E; Reichert Jung, Vienna, Austria) and stained with toluidine blue. Toluidine blue-stained slides were viewed and photographed with a Biorevo BZ-9000 microscope. Ultrathin sections of the tissue were cut, stained with uranyl acetate and lead citrate, and viewed with a Transmission electron microscope (Jeol JEM-1400Plus).

## 5. Conclusions

The novel congenic mouse strain *Tff3^−/−^*/C57BL6NCrl is a valuable model for assessing the role of Tff3 deficiency on liver disease relevant pathways. Here presented data show that *Tff3* deficiency is not crucial for acute ERS response caused by Tm. The impact of *Tff3* deficiency in the liver can be seen at the level of the transcriptional regulation of immune response relevant genes. 

Since liver acts as an important metabolic and immunological organ, the influence of *Tff3* deficiency and physiological function possibly reflects upon the whole organism. However, this has to be determined in future investigations. 

## Figures and Tables

**Table 1:** Oligonucleotides used for qPCR analysis.

Gene Symbol	Accession No.	Primer Sequence Forward (5′-3′) Reverse (5′-3′)	Optimized qPCR Conditions (Annealing Temp/MgCl)
ERS markers			
*ATF4*	NM_009716.3	CCACTCCAGAGCATTCCTTTAG CTCCTTTACACATGGAGGGATTAG	59 °C; 3.5 mM
*BIP*	NM_001163434.1	GAGACTGCTGAGGCGTATTT CAGCATCTTTGGTTGCTTGTC	58 °C; 3.5 mM
*CHOP*	NM_007837.4	TTGAGCCTAACACGTCGATTAT CACTTCCTTCTGGAACACTCTC	58 °C; 3 mM
*EDEM*	NM_138677.2	TGAAAGCATGTGAGGGTAGTG GAGAGAAGGGAAGACAGGATAGA	61 °C; 3.5 mM
*GRP94*	NM_011631.1	AAGAATGAAGGAAAAACAGGACAAAA CAAATGGAGAAGATTCCGCC	58 °C; 3 mM
*sXBP1*	NM_008934.4	GAGTCCGCAGCAGGTG GTGTCAGAGTCCATGGGA	56 °C; 3 mM
Cytokines			
*CXCL1*	NM_008176.3	GTGTCAACCACTGTGCTAGT CACACATGTCCTCACCCTAATAC	61 °C; 3.5 mM
*IL1α*	NM_010554.4	CCTTACACCTACCAGAGTGATTT CCTTACACCTACCAGAGTGATTT	65 °C; 3 mM
*IL1β*	NM_008361.4	ATGGGCAACCACTTACCTATTT GTTCTAGAGAGTGCTGCCTAATG	64 °C; 3 mM
*IL6*	NM_031168.2	GATAAGCTGGAGTCACAGAAGG TTGCCGAGTAGATCTCAAAGTG	59 °C; 3.5 mM
*MCP1*	NM_011333.3	CCTGGATCGGAACCAAATGA CGGGTCAACTTCACATTCAAAG	62 °C; 3 mM
*TGFα*	NM_031199.4	CTTTAGGAAGGACCTGGGTTG GTGTGTCCAGGCTCCAAATA	66 °C; 3 mM
*TNFα*	NM_013693.3	GTCTCAGAATGAGGCTGGATAAG CATTGCACCTCAGGGAAGAA	63 °C; 2.5 mM
Oxidative stress markers			
*COX1*	NM_008969.4	GTGCCAGAACCAGGGTGTCT GTAGCCCGTGCGAGTACAATC	58 °C 3 mM
*GPX1*	NM_008160.6	GGTTCGAGCCCAATTTTACA CATTCCGCAGGAAGGTAAAG	58 °C 2.5 mM
*NOX2*	NM_007807.5	ACTCCTTGGGTCAGCACTGG GTTCCTGTCCAGTTGTCTTCG	62 °C 3 mM
*SOD1*	NM_011434.2	GCCTTCTGCTCGAAGTGGAT GGAAGCATGGCGATGAAAGC	59 °C 3.5 mM
*SOD3*	NM_011435.3	TGGCTGATGGTTGTACCCTG TGAGAAGATAGGCGACACGC	60 °C 2.5 mM
Housekeeping genes			
*ACTB*	NM_007393.5	GCAAGCAGGAGTACGATGAG CCATGCCAATGTTGTCTCTT	61 °C; 3.5 mM
*B2M*	NM_009735.3	CCTGCAGAGTTAAGCATGACAGT TCATGATGCTTGATCACATGTCT	60 °C; 3 mM

## Supplementary Materials

Supplementary materials can be found at https://www.mdpi.com/1422-0067/20/18/4389/s1.

## Abbreviations

ACTB	actin beta
ATF4	activating transcription factor 4
BIP	endoplasmic reticulum chaperone bip
B2M	beta-2-microglobulin
CHOP	DNA damage-inducible transcript 3
COX1	cyclooxygenase I
CXCL1	C-X-C motif chemokine ligand 1
eIF2α	eukaryotic translation initiation factor 2α
EDEM1	ER degradation enhancing alpha-mannosidase like protein 1
ER	endoplasmic reticulum
ERS	endoplasmic reticulum stress
GSTP1	glutathione S-transferase pi 1
GSTP2	glutathione S-transferase pi 2
GPX1	glutathione peroxidase 1
GRP94	heat shock protein 90 beta family member 1
IL1α	interleukin 1 alpha
IL1β	interleukin 1 beta
IL6	interleukin 6
MCP1	monocyte chemoattractant protein-1
MT1	metallothionein-1
MT2	metallothionein-1
NOX2	NADPH oxidase 2
PERK	protein kinase R (PKR)-like endoplasmic reticulum kinase
SOD1	superoxide dismutase 1
SOD3	superoxide dismutase 3
sXBP1	spliced X-box binding protein 1
Tffs	trefoil factor family proteins
TNFα	tumor necrosis factor alpha
TGFα	tumor growth factor alpha
Tm	tunicamycin
UPR	unfolded protein response
Wt	wild type
